# A facile and comprehensive algorithm for electrical response identification in mouse retinal ganglion cells

**DOI:** 10.1371/journal.pone.0246547

**Published:** 2021-03-11

**Authors:** Wanying Li, Shan Qin, Yijie Lu, Hao Wang, Zhen Xu, Tianzhun Wu

**Affiliations:** 1 Shenzhen Institutes of Advanced Technology, Chinese Academy of Sciences, Shenzhen, China; 2 University of Chinese Academy of Sciences, Beijing, China; 3 Shenzhen Shekou People’s Hospital, Shenzhen, China; 4 Shenzhen Aier Eye Hospital, Shenzhen, China; National Eye Centre, UNITED STATES

## Abstract

Retinal prostheses can restore the basic visual function of patients with retinal degeneration, which relies on effective electrical stimulation to evoke the physiological activities of retinal ganglion cells (RGCs). Current electrical stimulation strategies have defects such as unstable effects and insufficient stimulation positions, therefore, it is crucial to determine the optimal pulse parameters for precise and safe electrical stimulation. Biphasic voltages (cathode-first) with a pulse width of 25 ms and different amplitudes were used to *ex vivo* stimulate RGCs of three wild-type (WT) mice using a commercial microelectrode array (MEA) recording system. An algorithm is developed to automatically realize both spike-sorting and electrical response identification for the spike signals recorded. Measured from three WT mouse retinas, the total numbers of RGC units and responsive RGC units were 1193 and 151, respectively. In addition, the optimal pulse amplitude range for electrical stimulation was determined to be 0.43 V-1.3 V. The processing results of the automatic algorithm we proposed shows high consistency with those using traditional manual processing. We anticipate the new algorithm can not only speed up the elaborate electrophysiological data processing, but also optimize pulse parameters for the electrical stimulation strategy of neural prostheses.

## Introduction

Retinitis pigmentosa (RP) and age-related macular degeneration (AMD) are retinal degeneration (RD) diseases with a prevalence of about 1 in 4000 [1–3]. The photoreceptor cells of RP and AMD patients degenerate irreversibly and cannot convert optical inputs into neural spikes, resulting in the loss of part or even all visual functions. However, even for profound RD patients, some physiological structures and functions of the retinal ganglion cells (RGC) are usually still preserved [4, 5]. Therefore, the RGC can be stimulated to evoke the neural activity of the remaining retinal neurons [6–8], thereby providing a straightforward method for the blind to restore visual function.

Such retinal stimulation methods that have been used to restore visual functions include electrical retinal prosthesis [4, 9, 10], optogenetics [11], photochemistry [12], ultrasonic stimulation [13], etc. Among them electrical stimulation based retinal prosthesis have been well studied for decades and recognized as the mainstream for practical applications [14]. In recent years, such retinal prostheses have achieved great progress in the device safety, integration and miniaturization, including microelectrode arrays, stimulator, wireless circuits, chip packaging, and implantation surgery [15]. In addition, many clinical trials and commercial implantation have been reported [16–18]. New tools and stimulation strategies have also been reported [19–21] explore the relationship between RGC response and electrical stimulation parameters, which will provide a reference for obtaining the optimal pulse for safe and efficient visual perception.

Previous studies have used manual spike sorting with empirical rules to determine whether the neuron cells were responsive or not. For example, as the classical *ex vivo* electrophysiological tool, a microelectrode array (MEA) system was used to record the multi units of RGC electrical responses after the electrical stimulation, and some spike-sorting methods were used to convert a spike sequence containing multi-units activities into a single-unit spike sequence [22, 23]. Currently, a widely used spike-sorting software is Offline Sorter™ (Plexon Inc., USA) [24, 25]. However, this softwares has several critical drawbacks such as cumbersome manual data processing, tedious parameter setups, and long processing time. Although some automatic algorithms for spike-sorting have been proposed [26, 27], they can only perform spike detection and spike classification, without analyzing the electrical response of RGCs. For the optimization of electrical stimulation strategies, it is critical to understand the influence of the parameters, the distance between the electrode and the neuron, etc. Therefore, an algorithm needs to be developed to provide an effective tool for rationally evaluating the effects of various parameters on electrical stimulation.

Herein we propose a facile and comprehensive algorithm that can automatically perform the spike detection, the spike classification and identify the RGC electrical responses, and demonstrate their functions using MEA *ex vivo* results of wild-type (WT) mice. The RGC responses of three wild-type mice are calculated, and the percentage of responsive RGC units at different distances is analyzed to obtain an effective stimulation range. The relationship between RGC response and electrical pulse amplitude is also analyzed to determine the best electrical pulse amplitude for effective retinal stimulation.

## Methods

### Experiments

#### Sample preparation

**Fig 1:**
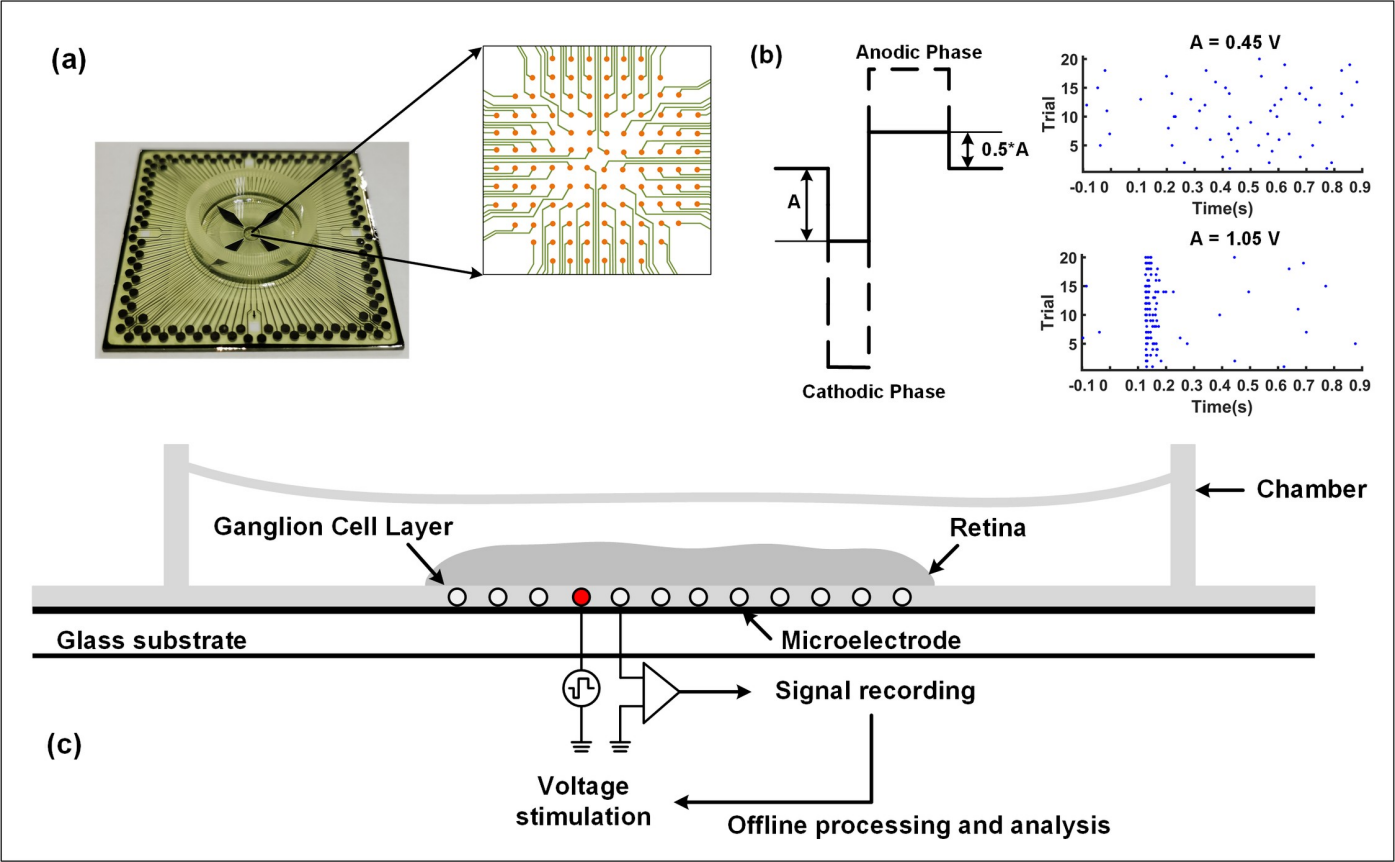
Experimental setup and illustration. (a) The MEA and its electrode layout. (b) A typical stimulation pulse with biphasic voltage and two typical RGC responses. When the cathode voltage is 0.45 V (the anode voltage is 0.225 V), the evoked spikes generated by RGC in 20 trials are scattering and irreverent. While for the 1.05 V amplitude, the evoked spikes have a clear cluster pattern. (c) Illustration of the experimental setup. One electrode performs electrical stimulation on the retinal ganglion layer (marked in red), and all other electrodes can record neural signals from the ganglion cells.

In this study, three 12-week-old C57BL/6J wild-type (WT) mice were used for the *ex vivo* experiment. The mice were dark adapted for more than 1 hour, deeply anesthetized by the injection of a mixture of ketamine (50 mg/kg) and xylazine (10 mg/kg). The eyeballs were removed under very dim red light, and the retina was peeled from the pigment epithelium in Ames’ medium (95% O_2_ and 5% CO_2_), finally cut into four parts. The ganglion cell layer was fixed upward, and attached to a filter membrane (Millipore, Cat. No. AABP02500) against the photoreceptor layer. The membrane was inverted onto a commercial MEA (120MEA100/30iR-ITO-gr, Multichannel System, Reutlingen, USA) (Fig 1). The animal experiments in this study had been approved by Institutional Animal Care and Use Committee of Shenzhen Institution of Advanced Technology, Chinese Academy of Sciences (approval number SIAT-IRB-180301-YGS-XUZHEN-A0244).

#### Electrical stimulation and recording

The MEA covered with retinal slices was connected to the MEA2100 data processing system and the MC_Rack software (Multichannel System, Reutlingen, USA) was used for electrical stimulations and signal recording from 120 electrodes. The sampling rate was configured to be 10 kHz/channel. The biphasic voltage pulses were always cathode-first, the voltage amplitude *A* for the cathode-side was from 0.15 to 1.65 V with a 0.15 V step, and the time duration *T* was constant (25 ms). Correspondingly, the anode pulse had a voltage amplitude of *A*/2 and a duration of 2*T* so that all injected charges were neutralized. The stimulation was repeated 20 times for all pulses of voltage, and the time interval between each stimulation was 10 s. In addition, the spontaneous activity trace of 0.1 s before stimulation and the induced activity trace of 1 s after stimulation were recorded, respectively. In this paper, a total of 3 WT mice retinas were stimulated with 17 center electrodes, and the response signals were recorded.

### Data processing

**Fig 2:**
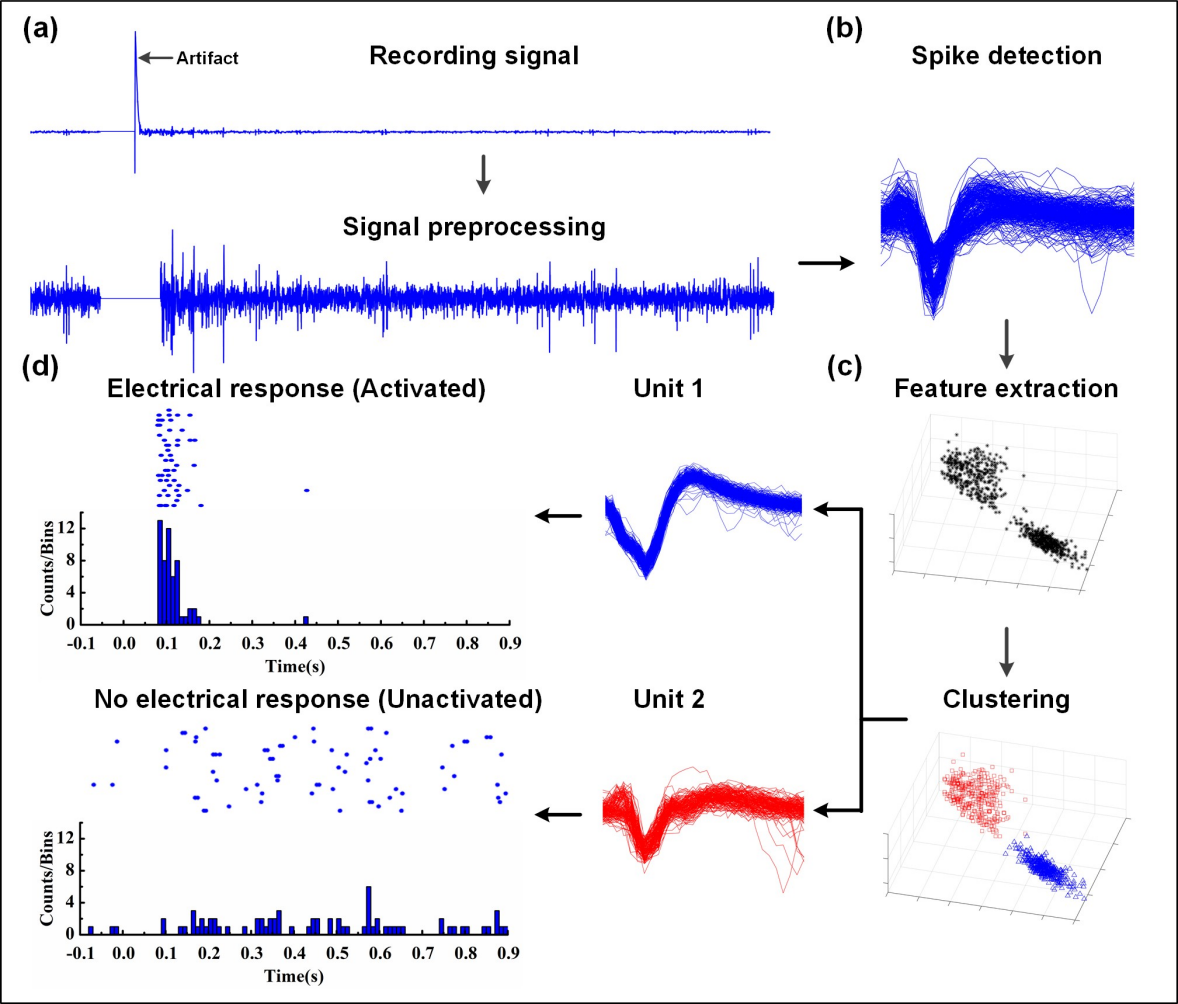
The processing flow of the proposed algorithm. (a) Remove artifacts from the raw data during the data preprocessing. (b) Spike detection, then a sliding window is used to obtain spikes of the same length. (c) The signal obtained is separated into single-units by feature extraction and clustering. (d) The response analysis to determine whether RGC units were activated or not after an electrical stimulation.

The major processes for the proposed algorithm to achieve spike-sorting and electrical response identification are shown in Fig 2, composed of preprocessing, spike detection, feature extraction and clustering, and electrical response identification. Each step is described as follows.

#### Signal preprocessing

Before spike detection, the activity signal train recorded needs to be preprocessed by removing the artifact (Fig 2(A)). Generally, there may be artifacts at the stimulus and the end of the recorded signal, whose amplitude is much higher than all spikes of neural responses. Therefore, the maximum amplitude of the signals for 120 channels is concentrated in a period of time, indicating that there are artifacts and reset them to zero.

#### Spike detection

**Fig 3:**
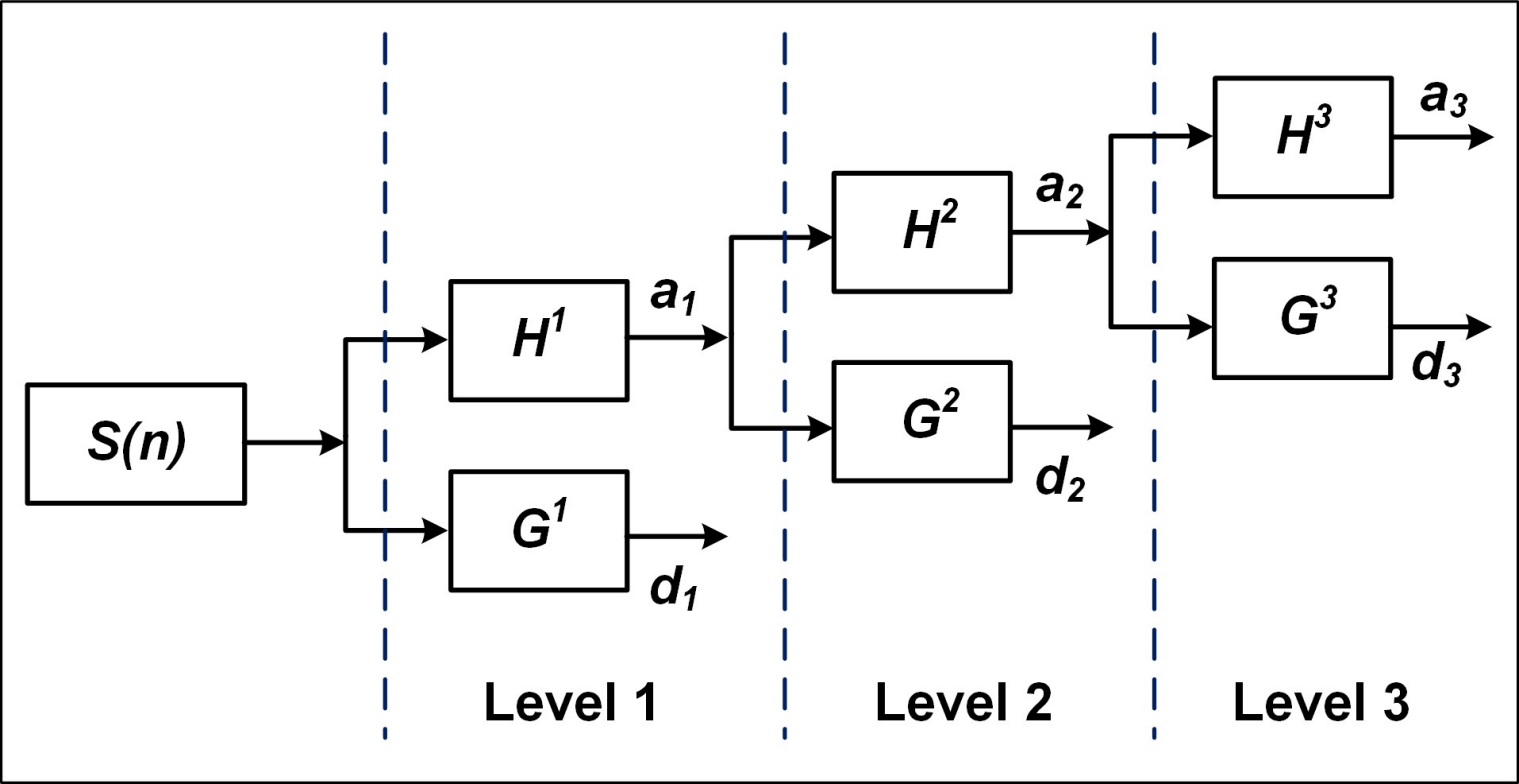
The tree structure of three-level decomposition with SWT. CA *a*_*1*_ and CD *d*_*1*_ are obtained by convolution of the signal *S* with HF *h*^*1*^ and LF *g*^*1*^, respectively. HF *h*^*2*^ and LF *g*^*2*^ (up-sampling of *h*^*1*^ and *g*^*1*^) are used to process *a*_*1*_ to acquire CA *a*_*2*_ and CD *d*_*2*_ of level 2. Finally, based on the up-sampling of *h*^*2*^ and *g*^*2*^, CA *a*_*3*_ and CD *d*_*3*_ of level 3 can be gained from *a*_*2*_.

Stationary wavelet transform (SWT) and Teager energy operator (TEO) were used for the spike detection (Fig 2(B)) [28], and their roles will be explained as follows. For SWT, the pre-processed signal is convolved with a high-pass filter (HF) and a low-pass filter (LF) to obtain the approximate coefficient (AC) and the detail coefficient (DC), respectively. Further, HF and LF filters are required for up-sampling (zeros are filled in adjacent data points) and are applied to AC, which can acquire other AC and DC [29]. In addition, SWT is data-redundant and translation-invariant, *i*.*e*., the length (time information) of the two coefficients is the same as the preprocessed signal, which can effectively reduce the adverse effects caused by the noise. The decomposition process of SWT is described by Eqs (1–2) and the three-level decomposition is illustrated in Fig 3.

$$a_{j+1}(k)=\sum_{n}h_{j}(n-k)a_{j}(k),$$ (1)

$$d_{j+1}(k)=\sum_{n}g_{j}(n-k)d_{j}(k).$$ (2)

Where *a*_*j*_ represents AC, *d*_*j*_ represents DC, *n* is the signal length, while *h*_*j*_ and *g*_*j*_ represent HF and LF, respectively.

To separate the spikes from the background noise and reduce the false detection rate, TEO is employed as the estimation of the transient energy of the signal. The spike transient behavior (the abrupt change in amplitude) shows higher instantaneous energy, so that the TEO coefficient of the spike is greater than the background noise [30]. For discrete signals, TEO can be expressed by Eq 3, where *x(n)* represents the signal amplitude at timestamp *n*:

$$\psi (x(n))=x^{2}(n)-x(n-1)x(n+1)$$ (3)

**Fig 4:**
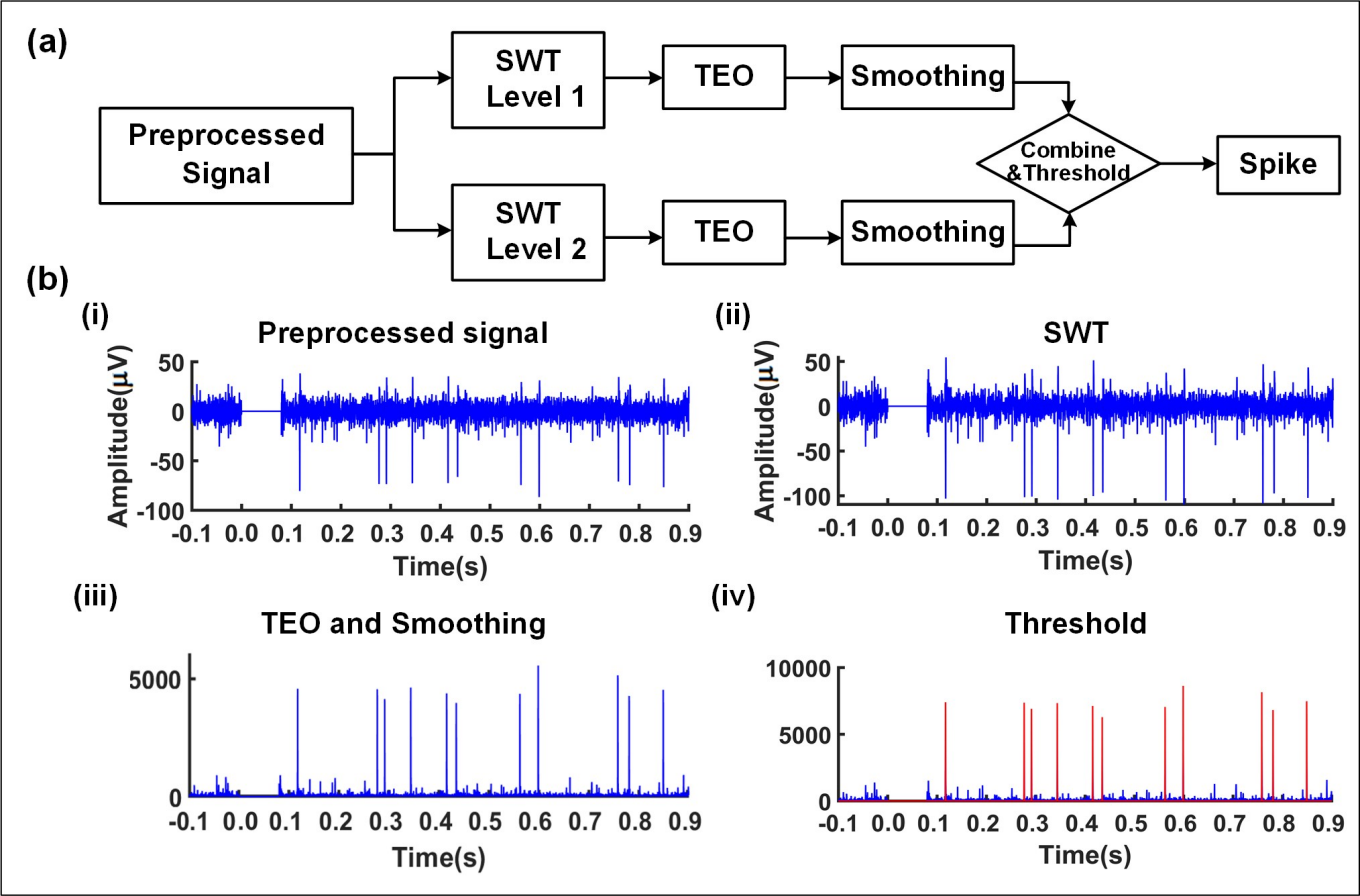
The processing algorithm for spike detection. (a) The flow chart of the algorithm based on SWT and TEO. (b) The typical processing results based on the algorithm. (i) Preprocessed result of the original signal. (ii) The amplitude of the spike increases after the level 1 of SWT processing. (iii) The TEO algorithm and window smoothing are used. The spikes become larger while the noises become smaller. (iv) The two-level output of SWT is processed by TEO and smoothing, respectively, and their corresponding results are combined. Then spikes (*red*) are detected using a given threshold.

The block diagram and corresponding results of the spike detection algorithm based on SWT and TEO are shown in Fig 4. A HF using Level 1 and Level 2 of SWT approximate coefficients is firstly applied, then TEO is applied to each frequency sub-band, and the Hamming windows are used to smoothen each independent convolution to eliminate spikes caused by the noise [28]. Finally, all sub-band are combined, and the spike detection process is completed through the comparison of the spike peaks with a given threshold.

**Fig 5:**
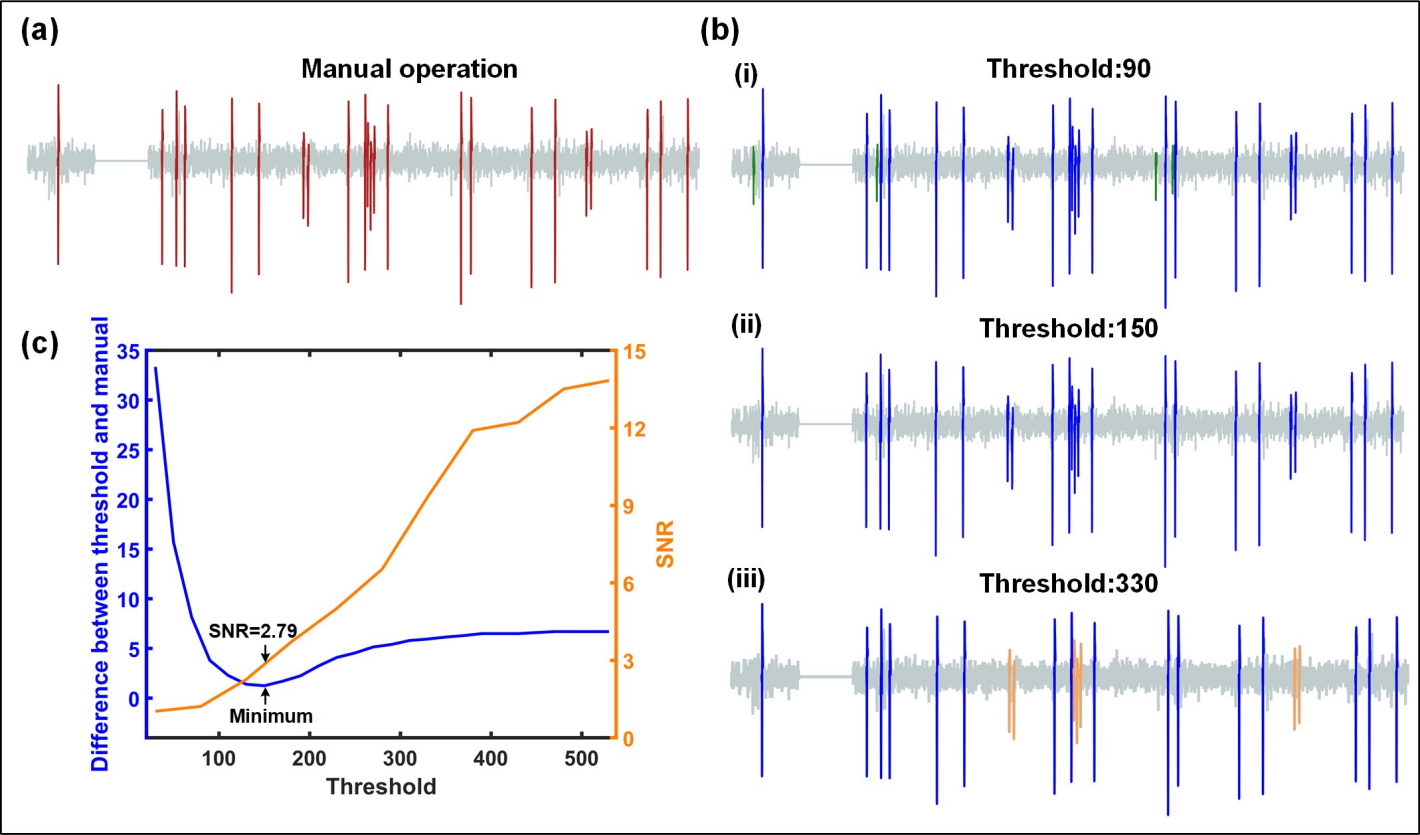
Determination of the optimal threshold. (a) The spikes (*red*) in the signal are marked out manually as RGCs responses. (b) The output results using different threshold values (*blue*) (i) When the threshold value is 90, there are 4 over-estimated spikes (*green*) in the detection result. (ii) Setting the threshold value to 150, all spikes are identical to those in Fig 5 (A). (iii) There are 6 under-estimated spikes (*orange*) for the threshold value of 330. (c) The peak number difference and SNR cost with different thresholds (the average of 20 trials for each threshold). In this case the threshold value 150 is corresponding to the lowest point of the curve, which is defined as the optimal threshold. When the SNR is lower than 2.79, false detection is prone to occur.

By setting different threshold values, we can achieve different spike detection results as shown in Fig 5. A small value of the threshold will over-estimate the spike peak numbers, while a larger one may result in under-estimation. Therefore, a proper threshold is critical. The threshold is obtained by comparing the results of detection algorithms and manual operations. The criterion for manual marking is that the spike amplitude is more than 2 times of the baseline bandwidth. There is a difference between the threshold setting and the result of manual judgment, and the relationship is described in Fig 5 (C). The result of manual marking is considered reliable, so the optimal threshold value is defined as the one with lowest difference. In addition, the signal-to-noise ratio (SNR) is an essential factor in the detection algorithm and is defined as Eq 4 in this study [31]. The SNR cost of changing thresholds (Fig 5(C)) shows that when the SNR is 2.79, the performance of the detection algorithm reaches optimal.

$$SNR=(\frac{peak‐to‐peak\;value\;of\;detected\;signal}{peak‐to‐peak\;value\;of\;noise\;segment}{)}^{2}$$ (4)

#### Feature extraction and clustering

**Fig 6:**
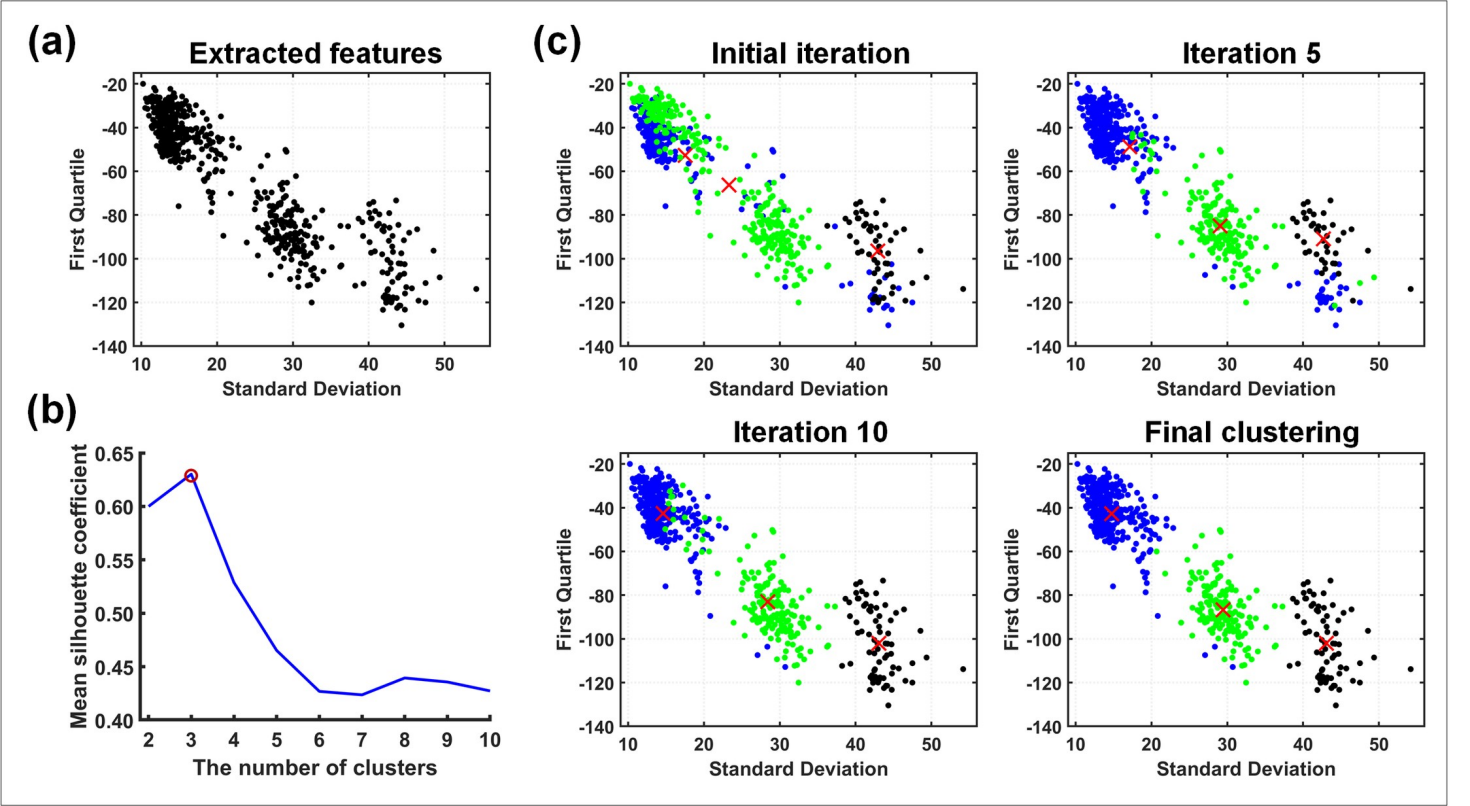
The extracted features are separated into single-unit. (a) The standard deviation and first quartile of the wavelet packet coefficients are displayed. (b) shows the relationship between the mean SC and the number of clusters, and the optimal number of K-means corresponds to the maximum value of mean SC. (c) The clustering process of the K-means method.

**Fig 7:**
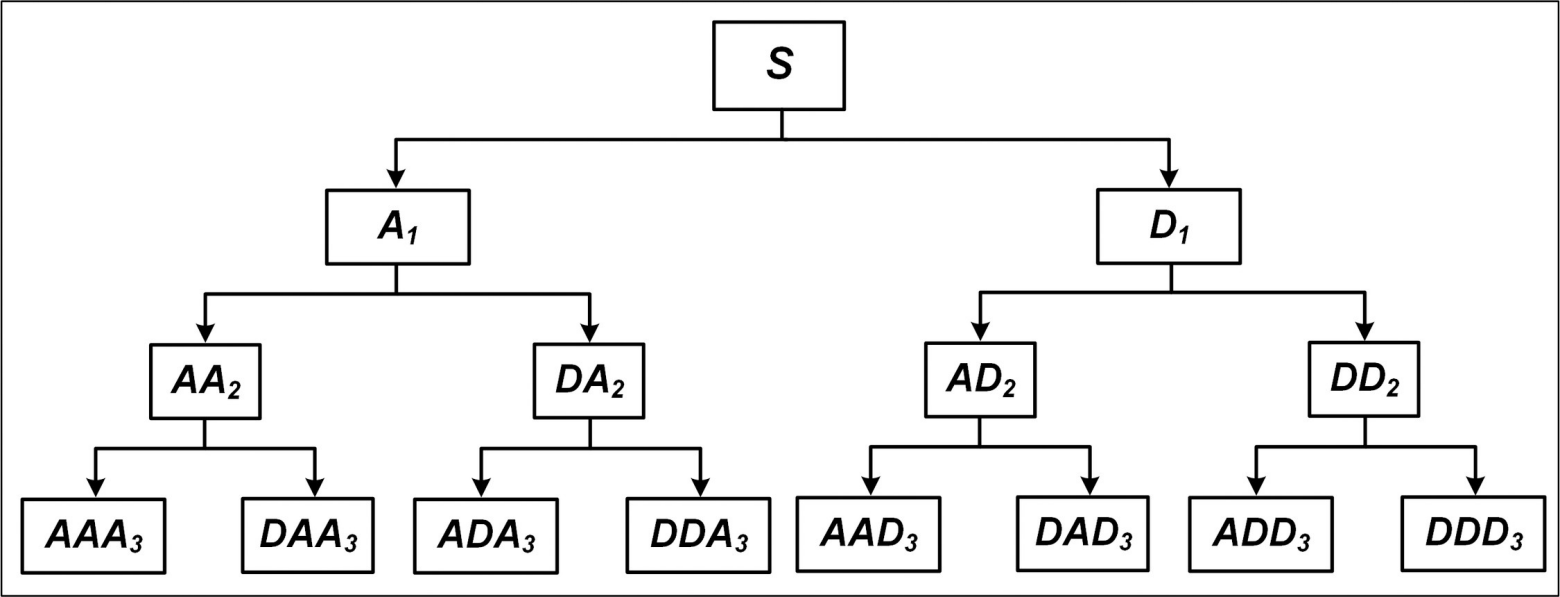
The three-level decomposition with WPD. *S* is the spike, while AC and DC are represented by capital A and D, respectively.

After the spike detection, feature extraction and clustering of the obtained spikes are required, the clustering iterative process of extracting features is shown in Fig 6. The method used for the feature extraction is wavelet packet decomposition (WPD), which has been used to analyze electroencephalography signals [32]. WPD is also a wavelet transform, but provides more frequency resolution than the discrete wavelet transform, *i*.*e*., both AC and DC are further decomposed [33]. Fig 7 shows the three-level decomposition with WPD, it is used to perform a new parameterized wavelet decomposition on the spike signal, and then the wavelet packet coefficients extracted from the five scales are used as the basis of clustering features [34], *i*.*e*., the K-means method [35] in our case.

The K-means method is based on conventional distance measurement by dividing the extracted wavelet packet coefficients into single-unit through clustering centers, and assigning each feature to a corresponding class. The algorithm is described in detail as follow:

1. K samples are randomly selected from the dataset of wavelet packet coefficients as the initial centroid of the K-means, *i*.*e*., {*μ*_1_,*μ*_2_,…,*μ_k_*}
2. Each sample is sorted to the cluster with the closest centroid. Further, the distance between the sample and the centroid is calculated by Eq 5:
  $$d_{ij}={\left\|x_{i}-\mu_{j}\right\|}_{2}^{2}$$ (5)
  Where *x*_*ij*_ represents a sample of dataset, when *d*_*ij*_ is the minimum value, *x*_*ij*_ belongs to the cluster of *μ_j_*.
3. The new centroid is obtained by recalculating the average of the samples in each cluster. It can be expressed by Eq 6, where *C*_*j*_ represents the cluster *j*:
  $$\mu_{j}=\frac{1}{\left|C_{j}\right|}\sum_{x\in C_{j}}x$$ (6)
4. Step (2) is returned to iterate, until the new centroid is the same as the centroid acquired in the previous iteration, this step is terminated and outputs the clustering results, *i*.*e*., {*C*_1_,*C*_2_,…,*C_k_*}.

Since the K-means method needs a manual determination of the cluster numbers, the Mean Silhouette Coefficient (SC) method [36] is used to solve this problem. For mean SC, the intra-cluster and inter-cluster distances can be used to evaluate the quality of the clustering results, and the process is illustrated as follow.

1. For each sample *i*, the average distance *a(i)* from all other samples in the same cluster is calculated.
2. The average distance between sample *i* and all samples in the external cluster is computed, and all of the external clusters are traversed. The smallest distance is denoted as *b(i)*.
3. The SC of sample *i* can be obtained by Eq 7, and the value is in the range of [–1, 1].
  $$s(i)=\frac{b(i)-a(i)}{\max\{a(i),b(i)\}}=\left\{ \begin{array}{ll} 1‐\frac{a(i)}{b(i)}, & a(i)<b(i)\\ 0, & a(i)=b(i)\\ \frac{b(i)}{a(i)}‐1, & a(i)>b(i) \end{array}\right.$$ (7)
4. The mean SC of the dataset is acquired by the average SC for all samples.

A large mean SC indicates a good performance of the clustering, therefore, the optimal number of clusters in K-means can be determined by maximizing the value of the mean SC.

#### Identification of RGC responses

**Fig 8:**
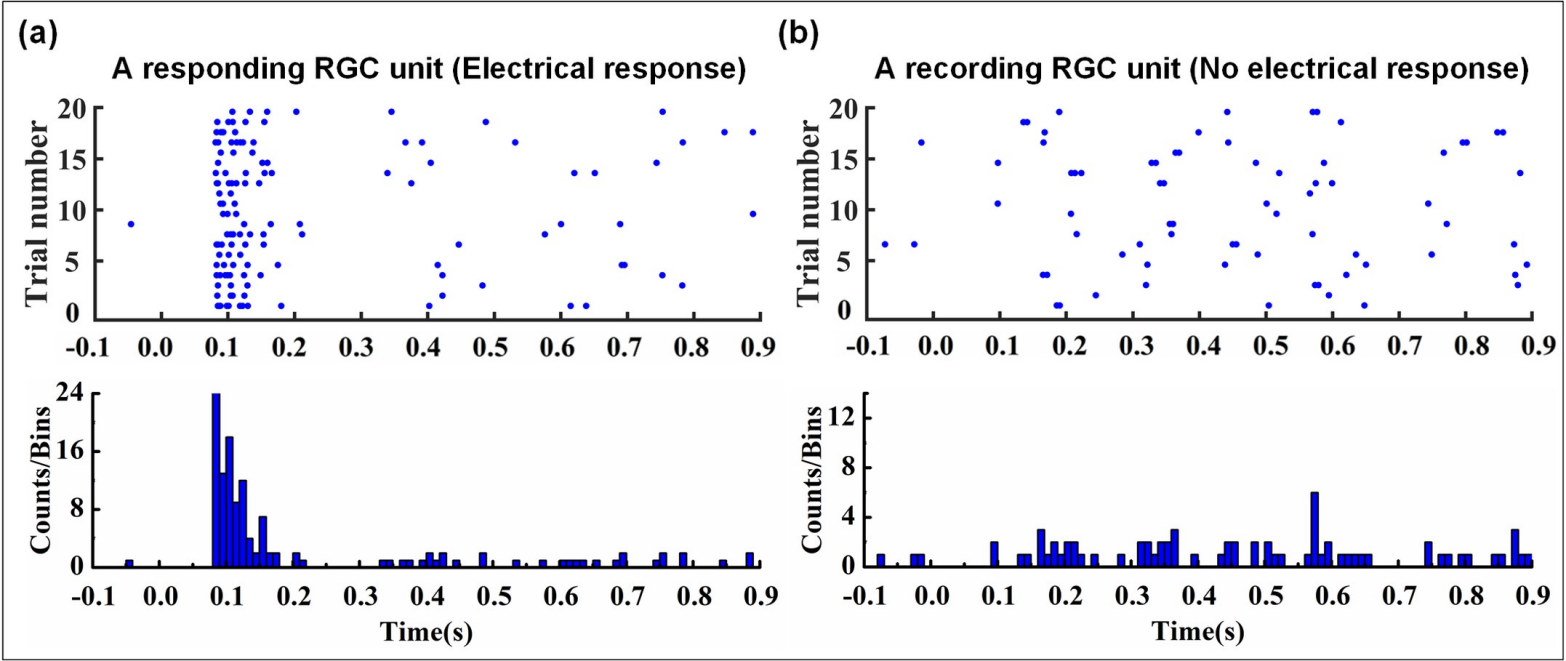
Identification of RGC responses. (a) The sample meeting the criteria is defined as an electrical response RGC unit. (b) The sample is inconsistent with the mentioned criteria and cannot be considered as a responding RGC unit. The raster plot is generated from the spike firing time of the clustered RGC units, and the total number of spikes in different time periods in the raster plot is displayed by the post-stimulus time histogram. In addition, -0.1s-0s: pre-stimulus time, 0s-0.9s: post-stimulus time.

After the clustering process the single-unit spike sequence was obtained. Then the raster plots (20 trials) and the post-stimulus time histogram (PSTH) of RCG responses can be also obtained. For WT mice, the criteria to define a responsive RGC unit should be met according to electrophysiological experiences: (1) The firing frequency within 300 ms after stimulation is more than 3 times of that within 100 ms before stimulation. (2) The RGC must meet criteria 1 for at least 10 times during the 20-time repetitive stimulation trials. According to these criteria, data from 20 trials are selected for the calculation (Fig 8).

### Data processing and analysis

As a comparison, some commercial softwares were used for the manual processing. The spike-sorting software Offline Sorter™ was used to process the same dataset offline to classify the action potentials into single units. In the Offline sorter™, the original signals were processed by low-cut filter, and the threshold was continuously adjusted and set to detect spikes for each channel. Based on PCA, the clustering algorithm was used to perform classification of the units. In addition, Spike2 (Cambridge Electronic Design Co., Ltd., UK) was used to complete offline data analysis (spike binning and alignment with stimuli), and Prism 7 (GraphPad Software, USA) was used for graphing [21]. Finally, the number of RGC units with and without electrical responses were manually counted and compared with the results automatically calculated by our proposed algorithm for validation.

In order to obtain the optimal pulse parameters for effective electrical stimulation, the relationship between the RGC responses and the input voltage amplitude is drawn and fitted using a graphing software Origin™ (OriginLab Corporation, USA). The number of evoked spikes from 20 trials and the corresponding voltages can be determined from the fitted curve.

## Results

### Statistical results of RGC electrical responses

The total and responsive RCG units (which describes the corresponding electric response intensity to the stimulation) obtained by our method and the commercial software are compared in Table 1. It can be seen that the sums of RGC units for the 17 stimulation trials are both 1193, while the total numbers of responsive RGC units in two results are 151 and 149, respectively. In addition, the maximum of all number difference using these two methods is only 3 which indicates a good consistence.

**Table 1:** The numbers of RGC units for our method and the manual operation software.

WT mice	Stimulation Trials	Our method		Manual processing	
		Total	Responsive	Total	Responsive
	No.1	44	9	43	11
	No.2	53	17	55	18
Sample 1	No.3	47	11	46	12
	No.4	44	17	45	14
	No.5	40	5	37	5
	No.1	98	11	97	12
	No.2	94	9	95	8
Sample 2	No.3	94	20	95	19
	No.4	96	15	96	13
	No.5	100	12	102	13
	No.6	93	3	92	3
	No.1	97	2	99	4
	No.2	66	3	69	2
Sample 3	No.3	58	4	55	5
	No.4	53	6	54	3
	No.5	58	3	57	5
	No.6	58	4	56	2
	Sum	1193	151	1193	149

### Characterization of RGC responses

**Fig 9:**
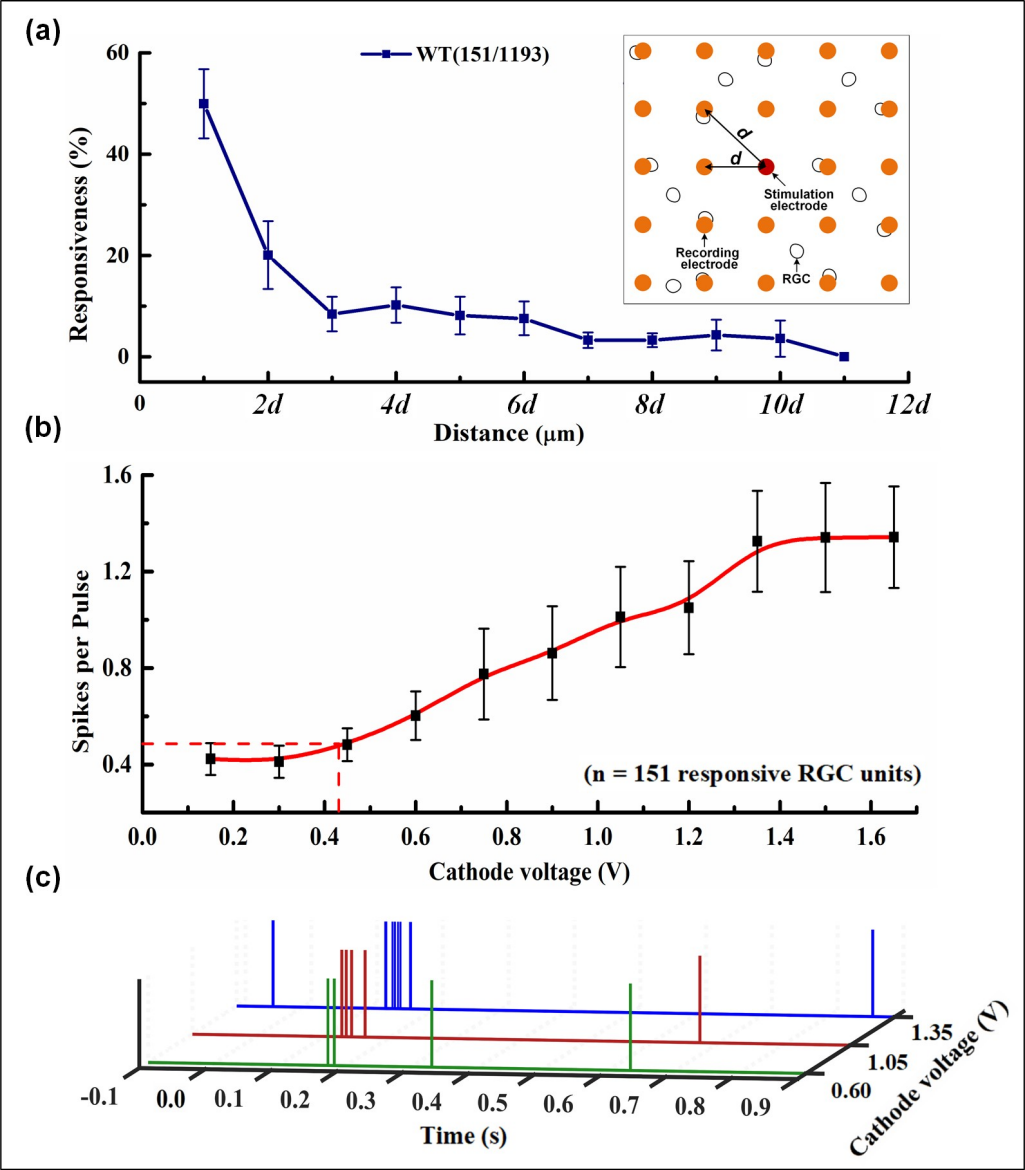
Characterization of RGC response. (a) The responsiveness percentage versus the distances from the stimulation electrode, where the distance between the stimulation electrode and the 8 electrodes adjacent to the first layer is regarded as *d*, and the second layer is 2*d*, etc. *d*∈ [100,141.4]. (b) The relationship between the average number of spikes and cathode voltage amplitude. 151 RGC units in total were calculated and the number of spikes were averaged for different stimulation voltage. (c) The time history illustration of spikes evoked by the responsive RGC unit upon different cathode voltages (0.60 V, 1.05 V and 1.35 V).

In order to analyze the relationship between the electrical responses and the distance from the stimulation electrode, the percentage of responsive RGC units among the total RGC units recorded at different distances between stimulating electrode and recording electrode is shown in Fig 9(A). Obviously, the number of responsive RGC units gradually decreases with the increasing distance, which agrees with previous studies [26, 27]. Specifically, when the distance is *d*, the percentage is 49.96%. As the distance increases to 3*d*, the percentage declines rapidly. The responses are fairly low when the distance exceeds 3*d*, and disappear for 11*d*. In this case, it can be considered that the electrical stimulation affects RGCs within a radius of 3*d*, *i*.*e*., this method can be used to characterize the influential range of electrical stimulation. In general, the retinal electrical stimulation becomes precise with the decreasing radius.

Fig 9(B) shows the relationship between the spike per pulse (defined as the average number of spikes within 300 ms after stimulation, which indicates the RGC response frequency) and the stimulation voltage amplitude (the cathode voltage is used as the typical case). The range of cathode voltage amplitude was from 0.15 V to 1.65 V, and the pulse duration was 25 ms. 151 responsive RGC units in total were measured. As seen, when the pulse amplitude raised from 0.45 V to 1.20 V, the average number of spikes increases monotonically until the voltage reached 1.35 V and it tends to be saturated. When the average number of spikes was 0.5, the cathode voltage amplitude was calculated to be 0.43 V according to the fitting curve, which was defined as the threshold voltage. Fig 9(C) shows an example of the spike sequences corresponding to the three voltage amplitudes. It further shows that the number of spikes will increase with increasing voltage.

## Discussions

Through the comparison in Table 1, the accuracy of our proposed method is verified for the RGC electrical response analysis. Compared with traditional methods, since this algorithm can automatically perform spike classification and electrical response identification, the data processing time is greatly reduced. Although Chung *et al*. also proposed an automatic method [37, 38], but their method can only complete spike detection and classification. The algorithm proposed in this paper has taken RGC electrical responses identification and practical applications one step further.

In spike detection, based on SWT and TEO, our method can significantly distinguish the spike and noise. Therefore, the fluctuation of threshold has a slight influence on the results of spike detection, and the optimal threshold obtained with experience is suitable for most of the signals in this work. In contrast, the method proposed by Garcia *et al*. cannot increase the distinction between spikes and noise, and the threshold needs to be adjusted and updated for each signal [39, 40]. From this perspective, our method is more efficient and robust.

In Fig 9(A), the intensity of the electrical responses of RGC decreases with increasing distance, and the neurons within 10*d* from the stimulation electrode can be evoked. Note that with a distance of *d*-3*d*, the electrical response percentage has the greatest decreasing slope, so further analysis within this distance range is important for electrophysiological studies. Archana *et al*. used a MEA with an electrode spacing of 200 μm which caused the difficulty to observe the RGC responses with rich details (eg, 100μm) [3, 41]. While in our experiment, the interelectrode spacing is 100 μm, which enables the analysis with more details.

In Fig 9(B), the threshold voltage and saturation voltage are 0.43 V and 1.35 V, respectively, and the number of evoked RGC spikes within this range increases linearly. Hence, it is concluded that the linear range for controllable stimulation is 0.43 V—1.35 V for WT mice. Yong *et al*. also proposed an optimal range of 0.55 V—1.0 V [42], which is similar to our conclusion. However, the pulse amplitude interval used in their experiment was 0.5 V, while is finer than our voltage interval was only 0.15 V and presents more details on the voltage influence on the electrical responses of RGCs.

The main limitation of this study is only analyzed the relationship between the pulse amplitudes and their electrical responses, yet other stimulation parameters such as duration, pulse shapes and so on need to be systematically explored. In addition, the low electrode density and small coverage area of commercial MEA make it impossible to record enough signals, and a larger and denser MEA will be required. Furthermore, the threshold is obtained empirically and an adaptive setting method is needed for the subsequent research. Nevertheless, given the many advantages in comprehensive electrical response analysis, we anticipate the algorithm can be a facile and useful tool to develop improved stimulation strategies based on more types of animals.

## Conclusion

In this paper, an algorithm is developed to characterize the influential range of electrical stimulation and determine the optimal electrical pulse amplitude for effective retinal stimulation. The main function of this method includes spike detection, classification and electrical response identification. The results obtained by our method are in good agreement with those using manual operation softwares, yet the processing time is greatly reduced. This algorithm can evaluate the stimulated influence of pulse parameters, electrode distance and other factors, which is critical to be understood in the optimization of electrical stimulation parameters for retinal prostheses. Due to its facile use and high efficiency, this method is powerful for optimizing such parameters, and has the potential to be expanded for other electrophysiological studies in neural stimulation.
